# Extracellular Vesicles in Alzheimer’s Disease: Friends or Foes? Focus on Aβ-Vesicle Interaction

**DOI:** 10.3390/ijms16034800

**Published:** 2015-03-03

**Authors:** Pooja Joshi, Luisa Benussi, Roberto Furlan, Roberta Ghidoni, Claudia Verderio

**Affiliations:** 1 CNR Institute of Neuroscience, via Vanvitelli 32, 20129 Milano, Italy; E-Mail: pooja.joshii87@gmail.com; 2 Molecular Markers Laboratory, IRCCS Istituto Centro San Giovanni di Dio Fatebenefratelli, 25125 Brescia, Italy; E-Mails: lbenussi@fatebenefratelli.it (L.B.); rghidoni@fatebenefratelli.it (R.G.); 3 Institute of Experimental Neurology, Division of Neuroscience, San Raffaele Scientific Institute, via Olgettina 60, 20132 Milano, Italy; E-Mail: furlan.roberto@hsr.it; 4 IRCCS Humanitas, via Manzoni 56, 20089 Rozzano, Italy

**Keywords:** Extracellular Vesicles, Aβ assembly, neurodegeneration, oligomeric Aβ, toxcicity

## Abstract

The intercellular transfer of amyloid-β (Aβ) and tau proteins has received increasing attention in Alzheimer’s disease (AD). Among other transfer modes, Aβ and tau dissemination has been suggested to occur through release of Extracellular Vesicles (EVs), which may facilitate delivery of pathogenic proteins over large distances. Recent evidence indicates that EVs carry on their surface, specific molecules which bind to extracellular Aβ, opening the possibility that EVs may also influence Aβ assembly and synaptotoxicity. In this review we focus on studies which investigated the impact of EVs in Aβ-mediated neurodegeneration and showed either detrimental or protective role for EVs in the pathology.

## 1. Introduction

Alzheimer’s disease (AD) is a progressive degenerative disorders characterized by memory loss and cognitive decline [1]. The main pathohistological findings in AD are the intracellular accumulation of neurofibrillary tangles, composed of an abnormally phosphorylated form of tau protein [2] and the accumulation of extracellular senile plaques consisting of aggregated amyloid-β (Aβ) peptides [3,4]. These observations led to propose Aβ peptides (Aβ1-42) and tau proteins (total-tau and phosphorylated tau) as potential cerebrospinal fluid (CSF) biomarkers for AD degeneration [5,6]. Tau neurofibrillary inclusions originate in the enthorinal cortex (EC) well before the appearance of clinical symptoms and gradually spread to anatomically connected hippocampal region and the neocortex in a prion-like fashion [7,8]. Similarly, accumulation of specific forms of Aβ can be responsible for the transynaptic spreading of amyloid pathology [4,9,10]. During the disease the amount of plaques and tangles increases and a correlation between tau pathology and disease progression has been demonstrated by several studies [11].

### 1.1. Amyloidogenic Processing of Amyloid Beta Precursor Protein and Toxicity of Soluble versus Insoluble Aβ Forms

Biologically, monomeric Aβ is produced via the sequential enzymatic cleavage of the transmembrane amyloid beta precursor protein (APP) by two proteases, β and γ secretases [12,13]. The discovery of the APP gene was followed by the identification of missense mutations—associated with familial AD (FAD) located in and around the Aβ region of APP and affecting the production or aggregation properties of Aβ peptides. Aβ1-40/42 have been the dominant research focus, but it is well known that *N*- and *C*-terminally truncated or modified forms of Aβ peptides also exist in human brain [14,15,16,17,18] and CSF (for review see [19,20]). More recently longer Aβ isoforms, like the Aβ1-43 peptide, are gaining attention for their high propensity to aggregate into neurotoxic oligomers: such specie has been reported to be enriched in the brain of individuals affected by FAD and sporadic AD [21,22,23,24].

Heterogeneity in Aβ peptides is due to γ-secretase, that cleaves APP at different positions [20] and to peptide modification mediated by glutamynil cyclase or by phosphorylation [25]. Aβ peptides and in particular peptide 1–42 very rapidly aggregate and form Aβ plaques in a complex multistep process, which involves formation of different amyloid species. More precisely, Aβ monomers first assemble into small soluble oligomers, which convert over time into protofibrils and subsequently into long insoluble mature fibrils [26]. Aβ fibrils are characterized by a typical beta sheet structure and form extracellular Aβ deposits, commonly known as plaques. Plaque deposition leads to recruitment and activation of microglia, the immune cells of the brain, which may cause a secondary damage to neurons and synapses [27,28].

Insoluble plaques are considered quite inert structures while soluble Aβ oligomers, present in the tissue surrounding the plaques, are highly neurotoxic and correlate with disease severity [29,30]. Consistently, growing evidence indicates that soluble Aβ oligomers but not insoluble fibrils bind to neuronal dendrites and mediate synaptic dysfunction and spine loss [31,32,33].

Binding of Aβ oligomers to neurons is mediated by different types of surface molecules. Among these, the p75 neurotrophin receptor, insulin receptor, NMDA and AMPA receptors, the Wnt receptor Frizzled, PrP^c^ [34] and glycospingolipid GM1 ganglioside, were recently proposed as the principal membrane target of Aβ oligomers [35].

### 1.2. Intracellular Aβ Processing and Trafficking

Several studies demonstrated that Aβ peptides can be formed in different subcellular compartments such as endoplasmic reticulum, Golgi, TGN, endosomes, lysosomes [36,37] and sorted to mutivesicular bodies (MVBs) (see paragraph 2.1). In addition, conformational targeting of intracellular Aβ oligomers revealed their pathological oligomerization inside the endoplasmic reticulum [38].

Accumulation of Aβ aggregates inside neurons is normally prevented by autophagy, which delivers potentially toxic Aβ aggregates to lysosomes [39]. Interestingly, autophagy may also control Aβ release into the extracellular space, as indicated by reduced plaque load in mice with autophagy deficits [40]. The mechanism behind reduced Aβ secretion has been recently defined *in vivo*: Aβ accumulates in the Golgi and is lowered in the multivesicular bodies (MVBs) of autophagy-deficient cells. This observation suggests that autophagy controls Aβ trafficking from the Golgi to MVBs and that Aβ secretion to some extent occurs via a mechanism involving MVBs [41]. 

### 1.3. Extracellular Vesicles (EVs) as Potential Modulators of Extracellular Aβ Assembly and Activity

Understanding and manipulating Aβ aggregation outside cells and interaction of soluble Aβ oligomers with neurons may provide key knowledge for treatment of AD. Despite massive efforts, how extracellular factors regulate the assembly and neurotoxic activity of Aβ species in AD brain is still largely undefined.

Extracellular Vesicles (EVs) are small membrane vesicles which bud from the plasma membrane (microvesicles (MVs) also called ectosomes) or result from exocytosis of multivesicular bodies (exosomes). EVs are important mediators of intercellular communication, as they transfer specific proteins, lipids, (micro)RNAs and DNAs between cells [42]. Because of their small size, some EVs can move from the site of discharge by diffusion and reach several biological fluids, such as blood, CSF, urine and synovial fluid, where EVs are emerging as clinically valuable markers of disease states [43]. An impressive progress has been recently made in the knowledge of the cellular and molecular mechanisms of EVs in the healthy and diseased brain but still many questions remain to be answered with respect to different aspects of EV function. 

EVs have been suggested as potential carriers in the intercellular delivery of misfolded proteins associated to neurodegenerative disorders, such as tau and Aβ in AD, α-synuclein in Parkinson’s disease (PD), SOD1 in amyotrophic lateral sclerosis (ALS) and huntingtin in Huntington’s disease (HD) [44,45,46]. However, intriguing data have been published on the role of EVs in AD. MVs and exosomes produced by distinct types of brain cells, including neuron, astrocyte and microglia, contain Aβ forms and interact with extracellular Aβ species (see below). Some components of the machinery to synthesize and degrade Aβ peptides, e.g., elements of the γ-secretase complex [47] and the insulin degrading enzyme IDE, which proteolyzes Aβ 1–42 and Aβ 1–40 [48], have been identified in EVs. In addition, specific surface molecules mediating interaction between EVs and Aβ have been identified [49,50,51]. However, how EVs influence the complex process of Aβ aggregation remains controversial and whether EVs promote or counteract the deleterious action of Aβ is still a matter of debate.

This review aims at summarizing and critically discussing recently reported *in vitro* and *in vivo* data on the effect of EVs on Aβ aggregation and neurotoxicity in order to encourage new studies to clarify this critical issue and to stimulate the exploitation of EVs in AD therapy.

## 2. EVs Change the Equilibrium between Soluble and Insoluble Aβ Species

### 2.1. Effects of Exosomes on Extracellular Soluble Aβ

In 2006, Rajendran and colleagues provided first evidence that (i) Aβ peptides are generated in early endosomes and sorted to multivesicular bodies (MVBs) in APP-expressing neuroblastoma cells and (ii) the fusion of MVBs with the plasma membrane mediates the release of exosomes loaded with Aβ. The observation that typical proteins of exosomes, such as alix, accumulate around plaques supported *in vivo* possible interaction between exosomes and Aβ [45,52]. Subsequent studies demonstrated that APP and other APP metabolites are secreted within exosomes in APP-expressing neuroblastoma, confirming that MVBs are essential organelles for APP metabolism [47,53,54,55]. Finally studies on exosomes isolated from AD patients and APP transgenic mouse brains demonstrated that exosomes are specifically enriched with APP *C*-terminal fragments, a source of Aβ peptides [55]. Collectively these studies indicate that Aβ can be encapsulated into neuronal exosomes to be released extracellularly.

Only a few years ago, Yuyama *et al.* examined possible effects of exosomes, derived from primary neuronal cells and neuronal cell line on the aggregation state of extracellular Aβ. By mixing a preparation of seed-free soluble Aβ 1–42 with exosomes the authors found a significant increase in fibril formation, as indicated by the thioflavin T assay [56]. Acceleration of fibrillization induced by exosomes promoted Aβ internalization by cultured microglia and subsequent Aβ degradation. Thus, binding of Aβ to neuronal exosomes might serve as a pathway to remove excessive extracellular Aβ levels. Incorporation of exosomes bound to Aβ into microglia were subsequently validated *in vivo* by showing that exosomes pre-injected into the hippocampus of APP_SweInd_ mice co-isolate with Aβ and the microglial marker Iba1 [50]. In the latter study, Yuyama and colleagues also provided some insights into the mechanism trapping Aβ to the exosomal membrane and promoting its assembly: using surface plasmon resonance analysis (SRP) they demonstrated that Aβ binds to exosomes through glycosphingolipid glycans present on the exosomal surface. Indeed, intact exosomes but not exosomes pretreated with EGCase, to cleave glycosphingolipid glycans, directly interacted with Aβ immobilized onto sensor chip.

A recent study from an independent laboratory confirmed the capability of exosomes produced by astrocytes to promote aggregation of seed-free soluble Aβ species on the vesicle surface [51]. ELISA quantification of Aβ aggregates isolated by centrifugation at 20,000× *g* in the presence of anti-ceramide antibodies suggested a critical role for the sphingolipid ceramide rather than glycosphingolipid glycans in Aβ aggregation induced by astrocytic exosomes. This finding may be consistent with the lower glycosphingolipid expression in exosomes released from astrocytes than neurons [57]. However, it is worth notice that exosomes derived from glial cells bind to Aβ with less efficiency than exosomes of neuronal origin. Hence, glycosphingolipids may strongly influence the affinity of exosomes for Aβ [57].

Similar experiments were performed by the group of Kim, using a mixture of different sized soluble Aβ species (Aβ-derived diffusible ligands, ADDLs), yielding bands on SDS-page corresponding to Aβ monomers, trimers and tetramers [49]. An *et al.* showed that exposure of exosomes derived by N2a cells or isolated from human cerebrospinal fluid to ADDLs induced a clear loss of Aβ monomers, as detected by western blot analysis, and promoted binding and immobilization of Aβ oligomers on the exosomal surface. Through elegant biochemical experiments they demonstrated that Aβ sequestration on neuronal exosomes depends on surface expression of PrP, a known Aβ receptor, which binds oligomers with high affinity [58]. Therefore, in addition to glycosphingolipids and ceramide, the GPI-anchored protein PrP^c^ accounts for Aβ-exosome interaction at the vesicle surface. Whether neuronal exosomes contain more PrP than exosomes derived from astrocytes and microglia is not known. Clarification of PrP expression in exosomes generated by distinct brain cell types will define whether PrP may influence, along with lipids, the different propensity of exosomes for trapping Aβ [57]. 

Interestingly, PrP, glycosphingolipids and ceramide are localized in raft domains [59], and through interaction with Aβ may also target intracellular amyloids to exosomes/MVs [60]. This sorting mechanism may be consistent with the proposed role of lipids raft in setting up platforms to concentrate into EVs protein destined to secretion [61,62].

The strong decrease in extracellular Aβ monomers detected by An and colleagues upon incubation of ADDLs with neuronal exosomes has been interpreted by the authors as the result of possible Aβ degradation by insuline degrading enzyme (IDE), which is among the proteolytic cargo of exosomes [63]. However, decrease in Aβ monomers might result from oligomerization and stabilization of oligomers on EVs membranes. Consistent with this possibility, it has been recently shown that Aβ oligomers show little stability in the brain’s aqueous compartments and are very rapidly sequestered on cellular membranes [35]. Whether monomers or Aβ oligomers bind to the vesicle membranes is, however, still controversial [35,50].

### 2.2. Effects of MVs on Extracellular Aβ Aggregates

The effect of EVs on conformational transition of aggregated rather than seed-free Aβ or ADDLs has been recently explored *in vitro*. Using the thioflavin T dye-binding assay for amyloid fibril detection, Joshi and colleagues reported that microglial MVs promote formation of soluble Aβ species from extracellular aggregates [60]. Confocal microscopy using fluorescently-labelled Aβ fibrils confirmed that incubation with MVs reduces the fibril size. Interestingly, lipids were identified as the active components of MVs responsible for solubilization of aggregated Aβ. This finding is consistent with previous evidence that natural lipids shift the equilibrium from insoluble toward soluble highly toxic Aβ species [64]. This study confirms the critical involvement of lipids in EV-Aβ interaction, as described above. In addition, a fraction of soluble Aβ species, generated in the presence of MVs, was shown to associate with MVs, as indicated by increased Aβ floatation on sucrose gradient upon addition of MVs. However, further work using antibodies selective for different Aβ species is required to unequivocally demonstrate which Aβ species interact with the surface of microglial MVs. 

The action of microglial MVs on seed free Aβ or ADDLs has not been analyzed yet. Neither the effects of exosomes derived from either neurons or glia on aggregated Aβ. Thus, while there is no doubt that EVs interact with Aβ species, it remains undefined whether MVs and exosomes may have opposite or similar effects on Aβ assembly.

## 3. Do EVs Attenuate or Promote Neurodegeneration

Recent studies indicate that EVs influence Aβ neurotoxicity. However, whether exosomes and other MVs increase or decrease the detrimental action of Aβ is a matter of debate. The fascinating hypothesis that EVs may constitute a prion-like mechanism for spreading of Aβ and tau protein [44,51,60,65,66,67] is indeed counterbalanced by evidence indicating that exosomes may act as scavengers of neurotoxic soluble Aβ species [49,50,57].

### 3.1. Protective Action of Exosomes against Synaptotoxic Aβ

Yuyama and coworkers has recently shown that continuous administration of exosomes derived from wild type neuroblastoma or primary neurons in the hippocampus ameliorates Aβ pathology and synaptic disfunction in APP_SweInd_ mice [50,57]. The beneficial action of exosomes is associated to a marked decrease in Aβ burden and to a significant rescue of synaptophysin immunoreactivity in AD mice. Neuroprotection has been ascribed to the capability of exosomes to trap Aβ and to promote its clearance by microglia, as previously described in culture [56]. Consistently, exosome production decreases in old AD mice, suggesting that downregulation of exosomes may be related to plaque deposition [57]. Based on these findings, Yuyama and colleagues proposed exosome administration as a novel therapeutic approach for AD, which may efficiently enhance Aβ clearance by microglia and prevent plaque deposition. However, the authors are aware that possible dysfunction in the phagocytic activity of microglia in the course of AD may facilitate Aβ spreading in association with exosomes rather than promoting its clearance [50].

A further evidence of a potential neuroprotective role of exosomes in AD comes from a study on mouse primary neurons over-expressing FAD-associated PS2 mutations. It has been demonstrated that the presence of PS2 mutations results in strongly reduced levels of cystatin C release in association with exosomes [54]. Our interpretation is that, in familial AD, a reduction of exosomal cystatin C, a neuroprotective growth factor as well as an anti-amyloidogenic protein [68], might result in an increased Aβ aggregation and neurodegeneration. If our hypothesis is correct, familial AD patients might also benefit from exosome administration. 

Protective action against AD has been also reported for exosomes released by mesenchymal stem cells (MSCs), a type of adult stem cells isolated from connective tissue. Exosomes secreted by MSCs carry enzymatically active neprilysin, the most important Aβ-degrading enzyme in the brain. After internalization in N2a cells, overproducing Aβ, exosomes decrease both intracellular and extracellular Aβ levels [69]. MSC-derived exosomes have been already given to a patient affected by a severe inflammatory disease under compassionate use [70], and might have therapeutic potential in multiple inflammatory and degenerative diseases.

Finally, possible therapeutic activity of exosomes from wild type N2a cells or healthy CSF has been indicated by An and colleagues. They showed that i.c.v. infusion of exosomes counteracts disruption of LTP induced by injection of soluble Aβ species in rats [49]. Direct sequestration of Aβ at the exosomal surface via PrP^c^, rather than enhancement of Aβ degradation or clearance by microglia, likely represents the mechanism underlying neuroprotection. However, further experiments are required to corroborate this hypothesis and define whether exosomes reduce Aβ binding to neurons. 

Collectively these studies support a protective role for exosomes produced by wild type neurons or MSCs against Aβ toxicity.

### 3.2. Detrimental Action of EVs in AD Pathology

To opposite conclusions came Dinkins and coworkers who recently analyzed the effects of the inhibitor of neutral sphingomyelinase GW4869, a known blocker of exosome secretion, in 5XFAD AD mice. They showed that i.p. injection of GW4869 decreases exosome concentration in serum and amyloid plaque formation [51]. Since exosomes stimulate Aβ aggregation [50,51,56] and Aβ aggregates are less efficiently cleared by glia, the authors concluded that reduced plaque load is caused by decreased exosome-induced Aβ aggregation and subsequent phagocytosis by microglia [51,71]. This interpretation could be true, but it is important to note that the exosomes produced in mice overexpressing APP contain substantial amounts of Aβ. Therefore inhibition of exosome secretion *per se* may lead to lowered extracellular Aβ levels and hence decreased Aβ load. In addition, it should be pointed out that the action of EVs on Aβ assembly *in vivo* may be far more complex than what observed on Aβ monomers *in vitro.* For example, our *in vitro* data, obtained with a mixture of aggregated and soluble Aβ forms, indicate that MVs promote solubilization of Aβ fibrils rather than aggregation [60]. Accordingly, two independent studies [50,69] recently revealed that exogenous administration of exosomes in the brain of AD mice causes a decrease in plaque deposition, playing against a pro-aggregating action of exosomes. Thus it remains controversial whether alteration of sphingolipid metabolism rather than inhibition of exosome secretion may account for protective effects of GW4869 in 5XFAD AD mice. 

Despite these considerations, neurotoxicity of EVs in AD is consistent with recent evidence, which associates MV production from microglia to neurodegeneration in dementia patients [60,67]. It has been recently observed that a large number of MVs of myeloid origin are present in the CSF from AD patients, which contain neurotoxic Aβ species [60]. Notably, the concentration of myeloid MVs positive correlates with levels of total tau and P-tau in the CSF, two classical markers of AD neurodegeneration [60]. In addition, there is a significant correlation between number of microglial MVs and atrophy of the hippocampus, the brain region with higher density of tau neurofibrillary inclusions in AD patients. Instead in patients with mild cognitive impairment, production of myeloid MVs correlates with microstructural damage to the white matter. As EVs, especially larger MVs, contain hyperphosphorylated oligomeric tau [72,73] in addition to neurotoxic Aβ [60], these findings support the hypothesis that reactive microglia shed harmful MVs which propagate damage to surrounding oligodendrocytes and neurons. Degeneration of projecting axons may mediate the diffusion of the pathogenic process from the initially involved limbic area both by contiguity and along white matter tracts, to the rest of the brain, thereby underlying prion-like propagation of AD pathology [67]. However, it is still unclear whether increased secretion of microglial MVs is the cause of the disease or response to the disease. Indeed microglia surrounding plaques may overproduce neurotoxic MVs in response to excessive Aβ phagocytosis when degradative pathways are saturated [60]. Interestingly, high concentration of myeloid MVs in CSF, by sequestering extracellular Aβ, may lower Aβ 42 level in CSF, which represents earliest biomarker of AD.

## 4. Conclusions

**Figure 1:**
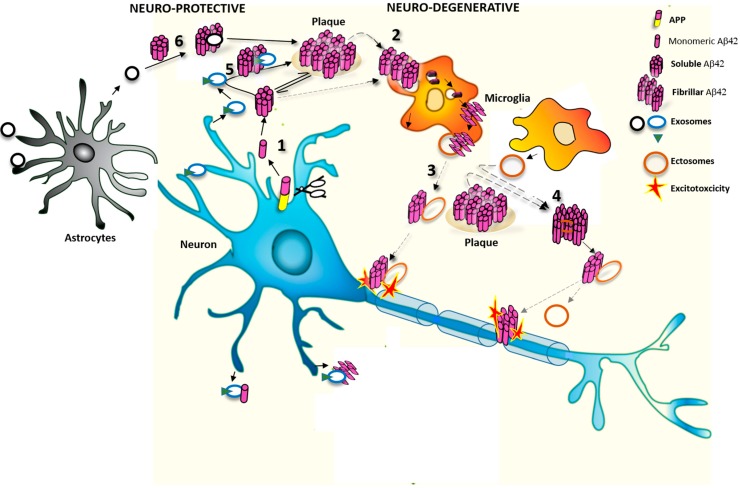
(**1**) Cleavage of APP leads to formation of monomeric Aβ42 forms, which aggregate to form soluble Aβ42 oligomers. Oligomers are then converted to insoluble fibrils, the main components of amyloid plaques; (**2**) Fibrillar and soluble Aβ42 species are internalized and degraded by microglia; (**3**) A fraction of internalized Aβ42 can be re-secreted as neurotoxic form, in association with microglial ectosomes [60]; (**4**) Microglia-derived ecotosomes also promote formation of soluble Aβ42 species from extracellular insoluble aggregates [60]; (**5,6**) In contrast to ectosomes, exosomes released by neurons or astrocytes promote aggregation of seed free soluble Aβ42 [51,56]. Neuronal exosomes may promote Aβ42 clearance by microglial cells [56].

Exosomes and MVs produced by distinct type of brain cells contain Aβ [45,53] and also interact with extracellular Aβ species through specific surface protein, such as PrP, and/or lipid components, *i.e.*, the sphingolipid ceramide and/or glycosphingolipids [49,50]. The overall effect of exosomes and MVs on extracellular Aβ levels and assembly may vary depending on vesicular Aβ content and type of parental cell (see Figure 1).

Neuron-derived exosomes, released by cells overproducing Aβ, likely represent a mechanism to get rid of excessive Aβ. Through exosome secretion neurons raise extracellular Aβ levels and promote Aβ aggregation. How exosome-mediated Aβ aggregation impacts on plaque load may crucially depend on Aβ phagocytosis and degradation by microglia. By contrast, neuron-derived exosomes containing normal Aβ levels and neuroprotective factors may act as scavengers for synaptotoxin Aβ species, thereby mediating neuroprotection [49,50,57]. In line with this hypothesis, the exosomal transport of cystatin C, a neuroprotective factor and an inhibitor of Aβ aggregation, is reduced in FAD [60]. Microglia-derived MVs also represent a way for microglia to eliminate neurotoxic Aβ when degradative pathways are saturated in response to excessive phagocytosis of amyloid plaque. However, Aβ storing MVs are toxic for neurons and oligodendrocytes and favor dissolution of extracellular Aβ aggregates, further increasing Aβ toxicity [60]. Thus, microglial MVs may seed and feed formation of neurotoxic amyloids throughout the brain, possibly representing the mechanism behind transynaptic spread of Aβ in AD. MVs production from microglia is very high in AD patients and correlates with classical markers of degeneration, white matter lesions and hippocampal atrophy, the best expression of neuronal damage in the human brain [67]. Further investigations are needed to better define the interaction of distinct EVs populations with different Aβ forms and their impact on Aβ assembly and cell-to-cell spreading.
